# Influence of Inflammatory State on the Need to Customize Parenteral Nutrition in Adolescents

**DOI:** 10.3390/nu16213782

**Published:** 2024-11-04

**Authors:** Jéssica Lavanholi Pinho, Renata Germano Borges de Oliveira Nascimento Freitas, Tiago Henrique de Souza, Roberto José Negrão Nogueira

**Affiliations:** 1 Department of Pediatrics, School of Medical Science, State University of Campinas (UNICAMP), Campinas 13083-887, Brazil; 2 Medical Clinical Department, State University of Campinas (UNICAMP), Campinas 13083-887, Brazil; nutrigene@uol.com.br

**Keywords:** customized parenteral nutrition, standardized parenteral nutrition, teenagers, inflammatory state, intensive therapy

## Abstract

Background/Objectives: Parenteral nutrition (PN) can be standardized or customized according to a patient’s individual needs, including clinical, metabolic, nutritional, and inflammatory conditions. The influence of inflammation on the indication of standard or customized PN for adolescents hospitalized in a quaternary hospital in the southeastern of Brazil was evaluated. Methods: A historical cohort study of 61 adolescents admitted to the hospital was conducted. Nutritional, clinical, and biochemical data from the first 7 days of PN use were analyzed. Elevated serum mineral and triglyceride levels, as well as renal or liver failure (grade III or IV), were considered unequivocal reasons for PN customization, while restoring energy-protein adequacy and low serum mineral levels were considered questionable reasons. Inflammatory status was analyzed during the study period. Results: A total of 128 PN solutions were prescribed, comprising 55 standardized and 73 customized. Overall, 40/61 patients required customized PN. The main reason for customization was to restore energy-protein adequacy (n = 48), while 24.7% (n = 18) of individualizations were for unequivocal reasons. Restoring energy-protein adequacy in the first 48 h was shown to have contributed to high transthyretin, which reduced the need for additional customized PN (r = −0.544; *p* = 0.044). A positive correlation was found between the total number of PN readjustments and C-Reactive Protein levels (r = 0.509; *p* = 0.044). Conclusions: Conditions such as malnutrition or an inflammatory state in adolescents presenting metabolic changes are indications for the use of customized PN.

## 1. Introduction

Parenteral nutrition (PN) is a therapeutic strategy [1] allowing intravenous administration of nutrients and may be standard or customized.

Pre-designed standard solutions use fixed macronutrient and electrolyte components available through multi-chamber bag solutions, and they are available in two forms: totally processed pre-mixed commercial formulations and where the compounding pharmacy supplying the institution produces several types of PN which, although only prepared on the day of use, are based on several pre-defined formulations [2]. In both cases, the composition is designed to meet the average needs of patients [3]. Thus, standard PN represents a viable option for health services that lack a proactive Nutrition Therapy Multi-Disciplinary Team (NTMT) [4]. Although posing an increased risk of both infection [4] and solution instability, some additional nutrition can be introduced to the PN solution [2,5,6]. Another option can be providing nutrients via an additional peripheral route.

The use of standard PN is more feasible in adults and adolescents without complicated disease conditions [7]. In fact, a European multi-center study showed that approximately 80% of standard PN was prescribed to adults [8]. However, the macronutrients and/or minerals contained in standard PN cannot be reduced, switched, or removed. Also, energy or aminoacidic improvements cannot be made to standard PN.

On the other hand, customized solutions can be adjusted to meet the patient´s individual nutritional needs [9]. These are dictated by age, weight, associated comorbidities, and by clinical, nutritional, inflammatory, and metabolic conditions [3]. Thus, customized PN would be the natural choice, particularly in age groups subject to variability in clinical conditions associated with diverse body composition, such as children and adolescents.

Moreover, a severe inflammatory status is very common among patients of quaternary hospitals, where the need for PN mainly coexists. It is known that, during the inflammatory process, the synthesis of acute phase proteins such as C-Reactive Protein (CRP) predominates, consequently reducing the synthesis of transthyretin (TTR), which is associated with sarcopenia [10,11].

Additionally, patients exhibiting inflammatory status may experience rapid systemic protein degradation and have a substantial protein requirement. Some advocate the use of amino acid infusion of up to 2.5 g/kg per day for critically ill adults [12].

Given that adolescents are an age group with specific needs, the objective of the present study was to assess the influence of an inflammatory status on the indication of standard or customized PN to adolescents hospitalized in a quaternary hospital in the southeast of Brazil.

## 2. Materials and Methods

### 2.1. Study Design and Protocol

A retrospective, longitudinal cohort study of adolescents (aged 10–19 years) hospitalized at the Clinics Hospital of the State University of Campinas (CH-UNICAMP)—a quaternary hospital in São Paulo (Brazil southeast region)—was conducted.

The data were obtained from patients’ individual evaluation records between 2012 and 2018. These records were devised prior to the study period for care and research purposes. Each record contains information used for clinical, nutritional, and metabolic follow-up of all hospitalized patients receiving PN.

The study inclusion criteria were age 10–19 years; use of PN either exclusively or as main source of energy (≥80% of nutritional needs); and hospitalized at the CH-UNICAMP during the established period. The exclusion criteria were use of peripheral PN and data registered on patient´s individual record deemed insufficient or inaccurate.

For the assessment of nutritional/metabolic status and inflammatory profile, data were retrieved for each patient from individual monitoring records holding information collected during treatment by the NTMT in the first 48 h of PN use; on the 4th day; and on the 6th or 7th day of PN use. These three periods were adopted due to the possibility of refeeding syndrome occurring in the first week of reintroducing nutrition to the patient.

### 2.2. Data on Hospital Stay and Nutritional Therapy

The following variables were analyzed: age (years); sex (male or female); type of hospital stay (intensive or ward); main diagnoses [post-operative gastrointestinal tract (GIT)]; post-operative non-GIT; short bowel syndrome (SBS); peri-operative inflammatory bowel diseases (IBDs); paralytic ileus of different causes; upper gastrointestinal hemorrhage (UGIB); pancreatitis with ileus; protein-losing enteropathy (PLE); chylous effusion/chylothorax; septic shock with ileus; polytrauma with ileus; Hematopoietic Stem Cell Transplantation (HSCT), with mucositis and/or severe colitis; PN type (standard or customized); classification of nutritional status [Z-score for Body Mass Index (BMI)/age]; duration of PN (days); total number of readjustments (number of times a new prescription of customized PN was required); reasons for customizing PN [restoring protein-energy adequacy; hypertriglyceridemia (>400 mg/dL); renal failure; grade III or IV liver failure; hypokalemia; hyperkalemia; hypophosphatemia; hyperphosphatemia; hyponatremia; hypernatremia; hypocalcemia; hypercalcemia; hypomagnesemia; hypermagnesemia; “hypomineral” + “hypermineral”—when concomitant presence of minerals below and above reference levels, respectively)]; and clinical outcome (discharge or death). Minerals (serum electrolytes) included the following: potassium, phosphorus, sodium, calcium, and magnesium.

The diagnosis of renal failure and/or liver failure (with grade III or IV encephalopathy) was established by a specialist physician or by the physician attending the patient during the hospital stay. Renal failure was defined as a 50% increase in plasma creatinine within 7 days or a 0.3 mg/dL increase in plasma creatinine within 2 days or oliguria or Glomerular Filtration Rate (GFR) ≤ 60 mL/min per 1.73 m^2^, as recommended by the Kidney Disease Improving Global Outcomes (KDIGO, 2012) [13]. The criteria used for defining acute liver failure were International Normalized Ratio (INR) > 2 without encephalopathy or INR > 1.5 with encephalopathy grade 3 or above [14].

### 2.3. Prescribing Parenteral Nutrition

Prescriptions of the customized PN bags were carried out by one of the 2 physicians from the hospital NTMT. These bags were formulated with the aid of a specific computer software tool, which verifies the compatibility of the solution. The composition of the PN was prescribed based on the recommendations of the American Society of Parenteral and Enteral Nutrition (ASPEN) [15], prevailing at the time of each data extraction.

The type of PN solution (standard or customized) received by the patient was established, and the reason for customizing the solution, where applicable, was stipulated for all timepoints.

- Standard PN

Six standard central PN formulations were available in the hospital study. Three of these formulations were determined based on the bodyweight category. The available categories were 46–55 kg (1370 kcal and 59 g of amino acids), 56–65 kg (1650 kcal and 75 g of amino acids), and >65 kg (1950 kcal and 82.5 g of amino acids). The other standard PN formulas were high amino acids (1725 kcal and 100 g of protein); renal (795 kcal, 30 g of protein and low electrolytes); and plus magnesium (1715 kcal, 90 g of protein and high magnesium sulfate). The complete compositions of these standardized solutions are given in Appendix A (Table A1).

- Customized PN

This was prescribed for cases where standard PN could not be indicated or continued. Two types of reasons for customizing PN were established: unequivocal and questionable.

The unequivocal reasons were plasma mineral levels above reference values, hypertriglyceridemia, and kidney and/or liver failure (with grade III or IV encephalopathy). These were classified as indubitable reasons because restoring adequacy would require a reduction, switch in profile of amino acids, or full withdrawal of macronutrients and/or micronutrients—changes that are not possible when using a standard solution.

The reasons for customizing the PN classified as questionable were (1) restoring protein-energy adequacy, since this is achievable by infusing a lower volume of PN solution, and (2) plasma mineral levels below reference values, since these can be replaced parallel to PN.

### 2.4. Nutrition Assessment

Data for the parameters age, sex, weight, and height were retrieved to calculate Z-score for BMI/age using the WHO AntroPlus software program^®^ version 3.2.2. Nutritional status of adolescents was classified according to the recommendations set by the World Health Organization (WHO) [16], as follows: very underweight was defined for Z-score < −3; underweight for Z-score between −3 and −2; normal weight for Z-score between ≥−2 and ≤+1; overweight for Z-score between +1 and +2; obesity for Z-score between +2 and +3; and very obese as Z-score >+3.

Anthropometric evaluation of adolescent patients is routinely performed at the hospital by the nursing team as per the recommendations of the WHO [16] and Anthropometric Standardization Reference Manual [17]. Bodyweight was measured using electronic scales with a capacity of 2.5 kg–150 kg accurate to the nearest 100 g, whereas height was measured using a stadiometer accurate to the nearest 0.1 cm (device brand name Filizola^®^). In cases where anthropometric assessment could not be carried out, weight and/or height measurements taken by the legal guardian of the patient were used.

### 2.5. Laboratory Monitoring

Patients in use of PN were monitored via laboratory tests ordered as recommended by the American Society for Parenteral and Enteral Nutrition (ASPEN) [18]. Sample collections and lab work-up were performed by the Laboratory of Clinical Pathology of the CH-UNICAMP and with the results registered in the patient records of the NTMT.

**Figure 1:**
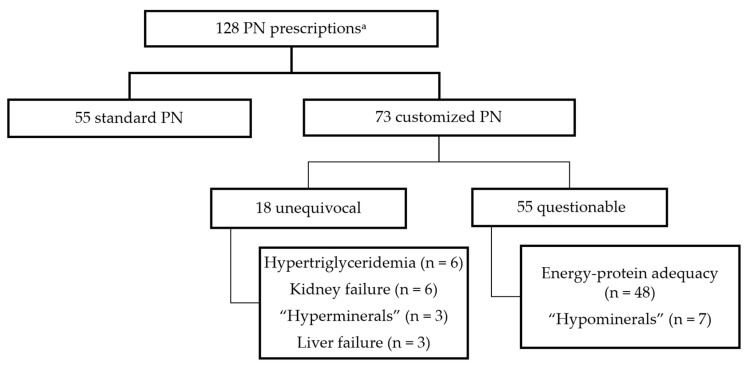
Flowchart of the total number of parenteral nutrition prescriptions during the first seven days of nutritional support. PN, parenteral nutrition. ^a^ Sum of all parenteral nutrition prescriptions in first 48 h, 4th day, and 7th day of use.

The assays and methods for determining levels to monitor patient inflammatory profile and their respective reference values were albumin [Colorimetric—bromocresol green (3.5–5.2 g/dL)]; HDL [Enzymatic Colorimetric-Direct (>45 mg/dL)]; C-Reactive Protein—CRP [Nephelometry (<10 mg/L)]; and transthyretin—TTR [Turbidimetry (20–40 mg/dL)]. The other assays, methods for determining concentrations and reference levels used for monitoring patient clinical and nutritional status, are provided in the Figure 1.

### 2.6. Statistical Data

The data held in the NTMT records were input into the Statistical Package for the Social Sciences^®^ (SPSS) version 22.0 for statistical treatment. Categorical variables were expressed as frequencies and continuous variables as mean (standard deviation—SD) or medians (IQR = p25 − p75). The Kolmogorov–Smirnov test was used to ascertain whether continuous variables adhered to a normal distribution. Parametric tests were used (Student´s *t*-test and Pearson´s correlation coefficient) for normally distributed variables, while non-parametric tests were conducted (Mann–Whitney and Spearman´s correlation coefficient) were used for non-normally distributed variables. The level of significance was adopted at 5%.

## 3. Results

Three records were excluded due to data inaccuracies, giving us a final sample of 61 patients. A total of 128 PN bags were prescribed (55 standard and 73 customized), and restoring energy-protein adequacy was the leading reason for customizing PN (48 events), followed by mineral imbalances (7 “hypomineral” and 3 “hypermineral” conditions), hypertriglyceridemia and renal failure (6 events each), and liver failure with grade III or IV encephalopathy (3 events). Of the 73 customized PN, 18 (24.7%) were prescribed for reasons considered unequivocal (Figure 1).

**Figure 2:**
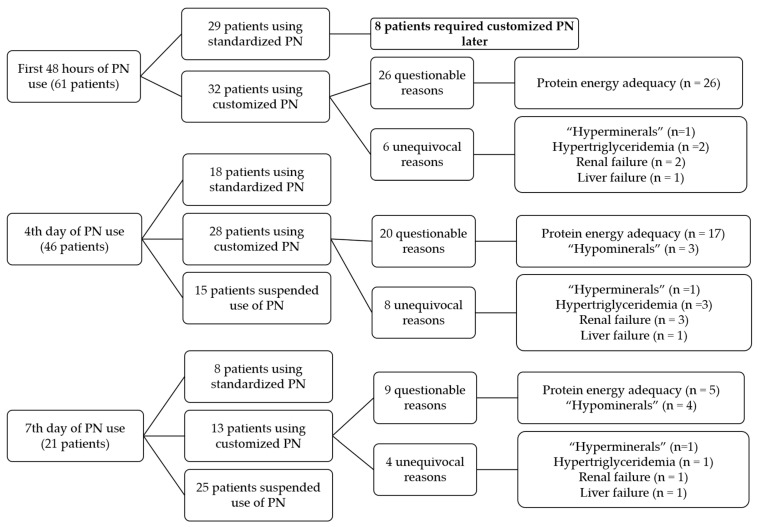
Distribution of patient groups receiving standardized PN and/or customized PN in first 48 h, 4th day, and 7th day of use. PN, parenteral nutrition.

Analysis of the first 48 h of PN use revealed that 32 patients received customized PN and 29 standard PN, although 8 of the patients in the standard PN group required customized PN later (Figure 2), i.e., 40/61 (65.6%) patients required customized PN at some point during the hospital stay.

Regarding characteristics of the sample, the adolescents [median age = 13.7 (IQR = 11.8–16.7) years] used PN as the main or only source of nutrition for a median time of 5 (IQR = 4–7) days. Generally, most of the patients were in intensive care (85.2%; n = 52) and had malnutrition (35.2%; n = 19). The leading reasons for the indication of PN post-operatively were for gastrointestinal tract and paralytic ileus (Table 1). No group difference was found in terms of sex, place of hospital admission, or clinical outcome (*p* > 0.05).

A statistically significant group difference was evident for age (*p* = 0.004), Z-score for BMI/age (*p* = 0.006), and CRP levels in the first 48 h (Table 2). The large differences between groups for certain variables such as transthyretin and CRP, especially for day 7, may have led to the failure to reach statistical significance; however, this may be explained by the low number of patients at this point in this study—after discontinuation of PN use. The data from the first 48 h and 4th day support the issue addressed regarding the inflammatory state. However, due to the decrease in sample data on the 7th day and the fact this was a historical cohort study, this conclusion could not be applied specifically at this timepoint of the analysis.

In the first 48 h of PN use, a higher Z-score for BMI/age correlated with a higher CRP and lower albumin levels. TTR level was positively correlated with albumin and HDL (Table 3). On the 4th day of PN, the higher the CRP level, the greater the need for PN readjustment. Conversely, the higher the TTR level, the lower the need for readjustment.

No statistically significant correlations were found on the 7th day of PN use.

## 4. Discussion

The results showed that patients with a more severe inflammatory state required a higher number of readjustments to PN during the hospital stay. Moreover, patients with higher TTR levels required fewer PN readjustments. Thus, the present study corroborated previous evidence showing a negative correlation between CRP and TTR [19]. An inverse correlation was found between TTR levels and the number of customized PN, suggesting that restoring energy-protein adequacy within the first 48 h contributed to metabolic/nutritional stability, a lesser need to readjust PN formulations, and, probably, to lower financial costs.

A total of 128 prescribed PN (55 standard and 73 customized) were assessed over a 7-day period. According to Z-scores for BMI/age, 35% of patients were malnourished. Assessing the nutritional status of critically ill patients can be hampered by limitations encountered in the hospital setting. Firstly, measuring height and bodyweight is difficult in bedridden patients due to sedation and/or insertions which limit mobility and, when readings can be taken, bodyweight may be overestimated due to the presence of fluid accumulation. In the present study, widely used biochemical markers such as albumin and TTR were assessed. During the inflammatory process, these proteins may be low due to inflammation rather than malnutrition, since the liver prioritizes the synthesis of acute phase proteins (CRPs), consequently reducing the synthesis of albumin and TTR [20,21,22,23].

Analysis of the sample in the first 48 h of PN use revealed that 40/61 (65.6%) patients with different ages and nutritional status required customized PN at some point. Patients with lower Z-scores for BMI/age needed more customized PN in the first 48 h, while most patients with higher Z-scores for BMI/age needed standard PN. With respect to age, standard PN was more frequently indicated for older adolescents. Given that the bodyweight of adolescents is closer to that of adults than children, it is plausible that standard PN met the nutritional needs of adolescents [24].

There was a direct correlation of Z-score for BMI/age with CRP and an inverse correlation with albumin. This finding may be due to an overestimation of patient bodyweight as a result of fluid accumulation, a common issue in critically ill patients. Consequently, the actual number of patients at a nutritional risk might be higher than that reported [25]. These findings corroborate the need to customize PN to restore energy-protein adequacy for some of the samples, particularly malnourished patients [26].

In the sample studied, adolescents predominantly had an active inflammatory status and were in an intensive care unit. This was also the case for patients with high CRP together with low albumin, TTR, and HDL. Patients with an active inflammatory status tend to need a larger amount of amino acids [12].

Overall, 55 customized PN solutions were prescribed to restore energy-protein adequacy and to fit plasma mineral levels. With the aim of energy-protein adequacy, customization can be justified to provide more protein. This is justified because patients with severe inflammation sometimes require more amino acids and fluid restrictions. In the hospital studied, the standard portfolio included a standard PN bag containing 100 g of amino acid (called high amino acid solution), but its use requires a considerable amount of fluid infusion. Therefore, this solution could lead to complications in patients with a severely impaired bodily fluid distribution. Consequently, the direct correlation of CRP levels with a need for a PN readjustment is logical, indicating that patients with a worse inflammatory state exhibit an impaired distribution of intracellular and extracellular compartments, rendering these individuals more unstable and requiring more readjustments in PN.

In cases of low plasma levels of minerals, these can be infused via an additional intravenous route separate from PN, although inserting multiple venous catheterizations or a multi-lumen catheter is not always possible. This option, as well as restoring energy and protein adequacy, is highly variable and should be discussed on a case-by-case basis. Thus, these reasons for customizing PN were deemed questionable, since the decision involves multiple variables and hinges on the capabilities of each health service. In any event, this decision cannot be made arbitrarily and should be based on the best possible medical practice and on the guidelines of the Society of Critical Care Medicine and American Society for Parenteral and Enteral Nutrition. These guidelines recommend that nutritional support should be customized according to the individual´s characteristics and dynamics of critical care, always considering the risk/benefit ratio [2].

A South Korean multicenter study [7] of adults and children (n = 70665) reported that 76.1% of patients aged > 15 years were prescribed standard PN. In the present study, 18 (24.7%) patients were prescribed customized PN for reasons considered unequivocal, similar to the rate reported in the South Korean study. An investigation conducted in France, Switzerland, and Belgium [8] reported that university hospitals prescribed more customized PN compared to non-university hospitals (78.1% versus 36.4%). It is likely that university, tertiary, or quaternary hospitals use more customized PN due to patient needs and due to the infrastructure available. Indeed, sites where human and institutional resources are lacking tend to avoid using customized PN because of the higher risk of pharmacokinetic errors and contamination [7].

The present study was carried out at a hospital with a proactive NTMT together with a computerized system. The key is the use of rigorous methods when prescribing and monitoring PN, coupled with an adequate pharmacy sterile compounding facility that ensures safe formulation [3,7,27]. Unfortunately, these facilities are not available in all hospitals. Given these issues, some services opt to use standard PN, even for severe inflammatory conditions [1].

In the present sample, standard PN could not be used in some cases (24.7% of PN solutions prescribed during first 7 days). These patients had renal or liver failure or hypertriglyceridemia or high plasma mineral levels. Failure to customize in these cases could be associated with a severe complication or even death. Irrespective of any of the other study findings, this scenario justifies having the option of customizing PN available across all hospital services, particularly where critically ill patients are involved. This level of nutrition support requires investment in human resources [28,29] with personnel qualified to handle cases under PN and the presence of a compounding pharmacy with the necessary infrastructure to ensure microbiological and pharmacokinetic safety [3,7,9,30]. In fact, personalized nutritional support contributes to a reduced rate of clinical complications, shorter length of hospital stay, and, consequently, to lower financial costs [31]. Thus, the nutrition protocol of health units should be tailored to their local context, considering factors such as target population, facilities, process limitations, and multidisciplinary experience [7,32,33].

## 5. Study Limitations

Given this was a cohort study, both patient follow-up and data registration were carried out by different physicians. Nevertheless, the service adopts a standard data collection record for clinical follow-up, where data were documented by specialists aware of the need to record this information. Moreover, the researchers participating in this study reviewed all the information during tabulation in an Microsoft Excel® version Professional Plus 2016 spreadsheet and omitted any inconsistent and/or insufficient data to minimize any inaccuracies.

Our sample size was limited; the sample, consisting of adolescents receiving PN, impedes generalizing our results. Given the small sample size, data could be analyzed as exploratory.

## 6. Conclusions

The current findings suggest that patients with a more severe inflammatory status required a higher number of readjustments. We also found that the restoration of energy-protein adequacy contributes to the metabolic stability of patients and reduces the need for later readjustments to PN.

Analyzing these data in adolescent inpatients of a quaternary hospital, especially those with an active inflammatory status, reveals that the use of customized PN, prescribed according to the specific needs of the patient, is an indispensable option in the presence of metabolic imbalances.

Other reasons for customizing nutrition should be carefully evaluated with regard to the risks and benefits of using standard PN solutions in adolescent patients that were originally formulated for use in adults.

## Figures and Tables

**Table 1:** Main characteristics of adolescents according to type of parenteral nutrition used in first 48 h.

	Standard PN (n = 29)	Customized PN (n = 32)	*p*-Value
Sex (n; %)			0.246
Male	17 (27.9%)	14 (23%)	
Female	12 (19.7%)	18 (29.5%)	
Admission (n; %)			0.602
Intensive	24 (39.3%)	28 (45.9%)	
Ward	5 (8.2%)	4 (6.6%)	
Diagnosis (n; %)			
Polytrauma	4 (6.6%)	2 (3.3%)	
Post-operative GIT	8 (13.1%)	14 (23%)	
Post-operative NGIT	0	2 (3.3%)	
Septic shock	2 (3.3%)	1 (1.6%)	
Paralytic ileus	9 (14.8%)	9 (14.8%)	
Hematopoietic Stem Cell Transplantation (HSCT)	1 (1.6%)	0	
Pancreatitis	4 (6.6%)	2 (3.3%)	
Protein-losing enteropathy	0	2 (3.3%)	
Chylothorax	0	0	
Short bowel syndrome	0	0	
Inflammatory bowel disease	1 (1.6%)	0	
Upper GI hemorrhage	0	0	
Nutritional status (n; %) ^a^			
Malnutrition	7 (13%)	12 (22.2%)	
Normal weight	8 (14.8%)	11 (20.4%)	
Overweight	11 (20.4%)	5 (9.3%)	
Clinical outcome (n; %)			0.129
Discharged	25 (41%)	31 (50.8%)	
Death	4 (6.6%)	1 (1.6%)	

^a^ Height data to classify nutritional status unavailable for 7 patients.

**Table 2:** Clinical, nutritional, and biochemical data for adolescents during use of parenteral nutrition.

	Patients with Standard PN ^a^	Patients with Customized PN ^a^	*p*-Value
Age	15.6 (12.6–17.4)	12.3 (11.3–14.5)	0.004
Days of parenteral nutrition	6 (4–7)	5 (4–7)	0.657
Z-score for BMI/age	−0.2 ± 2.04	−2.29 ±3.1	0.006
Biochemical assays			
● First 48 h of PN use			
Albumin	2.46 ± 0.94	2.70 ± 0.71	0.296
Transthyretin	10.4 (4.58–13.80)	8.47 (6.94–10.6)	0.680
C-Reactive Protein	85.2 (31.6–159)	12.3 (4.91–17.2)	0.003
HDL	20 (9–32)	23 (11–32)	0.266
● 4th day of PN use			
Transthyretin	11.43 ± 6.14	12.95 ± 6.32	0.668
C-Reactive Protein	81.4 (12.32–175)	27.5 (14.4–113)	0.536
HDL	21 (11–31)	17 (9.5–40.5)	0.879
● 7th day of PN use			
Transthyretin	15.9 ± 2.82	28.79 ± 14.5	0.198
C-Reactive Protein	18.25 (12.21–76.8)	54.3 (6.39–137)	0.788
HDL	18.5 ± 8.89	45.5 ± 13.44	0.038

Mann–Whitney or Student´s *t*-test. ^a^ Values expressed as mean ± standard deviation or median (p25–p75). Level of significance = *p* < 0.05. BMI = body mass index; HDL = high-density lipoprotein.

**Table 3:** Statistically significant correlations between clinical and nutritional markers.

Correlations	R-Value	*p*-Value	Classification
First 48 h of PN use			
Z-score for BMI/age and age	0.290	0.033	Moderate
Z-score for BMI/age and albumin	−0.359	0.012	Moderate
Z-score for BMI/age and CRP	0.686	0.000	Strong
Albumin and CRP	−0.643	0.001	Strong
Albumin and TTR	0.542	0.001	Strong
Albumin and HDL	0.624	0.000	Strong
TTR and HDL	0.404	0.018	Moderate
Fourth day of PN use			
Albumin and CRP	−0.599	0.018	Strong
Albumin and HDL	0.684	0.002	Strong
Albumin and TTR	0.811	0.001	Strong
Readjustments * and CRP	0.509	0.044	Moderate
Readjustments * and TTR	−0.544	0.044	Moderate
TTR and CRP	−0.817	0.007	Strong

BMI = body mass index; CRP = C-Reactive Protein; TTR = transthyretin; HDL = high-density lipoprotein. * Customized PN requiring readjustments over time.

## Data Availability

The raw data supporting the conclusions of this article will be made available by the authors upon request.

## Appendix A

**Table A1:** Compositions of standardized parenteral nutrition solutions at CH-UNICAMP.

	Basic 1 *	Basic 2 *	Basic 3 *	High Amino Acids Formula *	Renal *	Plus Magnesium *
Bodyweight (kg)	46–55	56–65	>65	-	-	-
Access route (vein)	Central	Central	Central	Central	Central	Central
Final volume (mL)	1650	1900	2250	2000	1000	2400
Calories (Kcal)	1370	1650	1950	1725	795	1710
Amino acids 6% (mL)	0	0	0	0	500	0
Amino acids 10% (mL)	590	750	825	1000	0	900
Protein (g)	59	75	82.5	100	30	90
Glucose 10% (mL)	0	0	0	0	0	0
Glucose 50% (mL)	420	500	600	450	250	500
Carbohydrates (g)	210	250	300	225	125	250
Long-chain triglycerides/Medium-chain triglycerides 20% (mL)	210	250	300	280	125	250
Lipids (g)	42	50	60	56	25	50
Non-protein Kcal/g nitrogen	120	112	123	83	140	94
Sodium chloride 20% (mL)	25	30	30	30	10	30
Potassium chloride 19.1% (mL)	20	20	20	24	0	20
Calcium gluconate 10% (mL)	20	20	20	24	10	20
Potassium phosphate (mEq)	12	15	18	16	0	15
Magnesium sulfate 10% (mL)	20	22	25	25	10	35
Multivitamin A (mL)	5	5	5	5	1,5	5
Multivitamin B (mL)	5	5	5	5	5	5
Trace elements (mL)	1.5	1.5	1.5	1.5	0.5	1.5
Distilled water (mL)	321.5	281.5	400.5	139.5	88	618.5

* Standard PN bags were formulated for teenagers and adults, requiring an adjustment of the total volume to meet needs of pediatric patients.

## References

[1] Ayers P., Adams S., Boullata J., Gervasio J., Holcombe B., Kraft M.D., Marshall N., Neal A., Sacks G., Seres D.S. (2013). A.S.P.E.N. Parenteral Nutrition Safety Consensus Recommendations. J. Parenter. Enter. Nutr..

[2] Boullata J.I., Gilbert K., Sacks G., Labossiere R.J., Crill C., Goday P., Kumpf V.J., Mattox T.W., Plogsted S., Holcombe B. (2014). ASPEN Clinical guidelines: Parenteral nutrition ordering, order review, compounding, labeling, and dispensing. J. Parenter. Enter. Nutr..

[3] Berlana D. (2022). Parenteral Nutrition Overview. Nutrients.

[4] Mihatsch W., Varas M.J., Diehl L.L., Carnielli V., Schuler R., Gebauer C., Marcos M.S.d.P. (2023). Systematic Review on Individualized Versus Standardized Parenteral Nutrition in Preterm Infants. Nutrients.

[5] Cartwright M.M. (2004). The metabolic response to stress: A case of complex nutrition support management. Crit. Care Nurs. Clin. N. Am..

[6] Deshmukh M., Grzejszczyk J., Mehta S., Patole S. (2018). Wastage of standardised parenteral nutrition solution—A challenge for neonatal units. J. Matern. Neonatal Med..

[7] Cheon S., Oh S.-H., Kim J.-T., Choi H.-G., Park H., Chung J.-E. (2023). Nutrition Therapy by Nutrition Support Team: A Comparison of Multi-Chamber Bag and Customized Parenteral Nutrition in Hospitalized Patients. Nutrients.

[8] Maisonneuve N., Raguso C.A., Paoloni-Giacobino A., Mühlebach S., Corriol O., Saubion J.L., Hecq J.D., Bailly A., Berger M., Pichard C. (2004). Parenteral nutrition practices in hospital pharmacies in Switzerland, France, and Belgium. Nutrition.

[9] Hamdan M., Puckett Y. (2024). Total Parenteral Nutrition. StatPearls.

[10] Linden M.A., Freitas R.G.B.d.O.N., Teles L.O.d.S., Morcillo A.M., Ferreira M.T., Nogueira R.J.N. (2024). Transthyretin and Nutritional Status in Critically Ill Adults on Parenteral Nutrition: A Prospective Cohort Study. Nutrients.

[11] de Figueiredo R.S., Nogueira R.J., Springer A.M., Melro E.C., Campos N.B., Batalha R.E., Brandão M.B., de Souza T.H. (2021). Sarcopenia in critically ill children: A bedside assessment using point-of-care ultrasound and anthropometry. Clin. Nutr..

[12] Yoshihara I., Kondo Y., Okamoto K., Tanaka H. (2023). Sepsis-Associated Muscle Wasting: A Comprehensive Review from Bench to Bedside. Int. J. Mol. Sci..

[13] Kellum J.A., Lameire N., Aspelin P., Barsoum R.S., Burdmann E.A., Goldstein S.L., Herzog C.L., Joannidis M., Kribben A., Levey A.S. (2012). Kidney disease: Improving global outcomes (KDIGO) acute kidney injury work group. KDIGO clinical practice guideline for acute kidney injury. Kidney Int. Suppl..

[14] Bansal S., Dhawan A. (2006). Acute liver failure. Indian J. Pediatr..

[15] American Society of Parenteral and Enteral Nutrition (2019). Appropriate Dosing for Parenteral Nutrition: ASPEN Recomendation. ASPEN Recommendations on Appropriate Parenteral Nutrition Dosing for Adult Patients. https://www.nutritioncare.org/pnresources/.

[16] World Health Organization (2006). WHO Child Growth Standards: Length/height-for-Age, Weight-for-Age, Weight-for-Length, Weight-for-Height and Body Mass Index-for-Age. Methods and Development.

[17] Norgan N.G. (1988). A Review of: “Anthropometric Standardization Reference Manual”. Edited by T. G. LOHMAN, A.F. ROCHE and R. MARTORELL. (Champaign, IL.: Human Kinetics Books, 1988.) [Pp. vi+ 177.] £28·00. ISBN 087322 121 4. Ergonomics.

[18] Canada T., Crill C., Guenter P., Canada T., Crill C., Guenter P. (2009). ASPEN Parenteral Nutrition Handbook.

[19] Gulhar R., Ashraf M.A., Jialal I. (2024). Physiology, Acute Phase Reactants. StatPearls.

[20] White J.V., Guenter P., Jensen G., Malone A., Schofield M. (2012). Consensus statement: Academy of Nutrition and Dietetics and American Society for Parenteral and Enteral Nutrition: Characteristics recommended for the identification and documentation of adult malnutrition (undernutrition). JPEN J. Parenter. Enter. Nutr..

[21] Krishnan K., Taylor M.D., Cresci G.A. (2015). Nutrition Assessment. Nutrition Support for the Critically Ill Patient: A Guide to Practice.

[22] Evans D.C., Corkins M.R., Malone A., Miller S., Mogensen K.M., Guenter P., Jensen G.L., the ASPEN Malnutrition Committee (2021). The Use of Visceral Proteins as Nutrition Markers: An ASPEN Position Paper. Nutr. Clin. Pract..

[23] Sotak Š. (2022). Inflammatory markers in clinical practice. Vnitr. Lek..

[24] Colomb V. (2013). Commercially premixed 3-chamber bags for pediatric parenteral nutrition are available for hospitalized children. J. Nutr..

[25] Lintz V.C., Vieira R.A., Carioca F.d.L., Ferraz I.d.S., Silva H.M., Ventura A.M.C., de Souza D.C., Brandão M.B., Nogueira R.J.N., de Souza T.H. (2024). Fluid accumulation in critically ill children: A systematic review and meta-analysis. eClinicalMedicine.

[26] Golucci A.P.B.S., Marson F.A.L., Ribeiro A.F., Nogueira R.J.N. (2018). Lipid profile associated with the systemic inflammatory response syndrome and sepsis in critically ill patients. Nutrition.

[27] Cogle S.V., Ayers P., Berger M.M., Berlana D.E., Wischmeyer P., Ybarra J., Zeraschi S., De Cloet J. (2024). Parenteral nutrition in the hospital setting/short-term parenteral nutrition. Am. J. Health Pharm..

[28] Berlana D., Almendral M.A., Abad M.R., Fernández A., Torralba A., Cervera-Peris M., Piñeiro G., Romero-Jiménez R., Vázquez A., Ramírez E. (2018). Cost, Time, and Error Assessment During Preparation of Parenteral Nutrition: Multichamber Bags Versus Hospital-Compounded Bags. J. Parenter. Enter. Nutr..

[29] Cogle S.V., Martindale R.G., Ramos M., Roberti G.J., Roberts P.R., Taylor K., Sacks G.S. (2020). Multicenter Prospective Evaluation of Parenteral Nutrition Preparation Time and Resource Utilization: 3-Chamber Bags Compared With Hospital Pharmacy–Compounded Bags. J. Parenter. Enter. Nutr..

[30] Choe J.H., Baek J.H., Jo Y.H., Cho Y.S. (2018). Infection Control in Parenteral Nutrition Preparation and Compounding. J. Clin. Nutr..

[31] Gramlich L., Kichian K., Pinilla J., Rodych N.J., Dhaliwal R., Heyland D.K. (2004). Does enteral nutrition compared to parenteral nutrition result in better outcomes in critically ill adult patients? A systematic review of the literature. Nutrition.

[32] Kozeniecki M., McAndrew N., Patel J.J. (2015). Process-Related Barriers to Optimizing Enteral Nutrition in a Tertiary Medical Intensive Care Unit. Nutr. Clin. Pract..

[33] Hill A., Elke G., Weimann A. (2021). Nutrition in the Intensive Care Unit—A Narrative Review. Nutrients.

